# Immune Checkpoint Blockade in Chinese Patients With Hepatocellular Carcinoma: Characteristics and Particularity

**DOI:** 10.3389/fonc.2022.764923

**Published:** 2022-03-10

**Authors:** Yaoqiang Wu, Han Lin, Xia You, Taiyan Guo, Tingting Sun, Hao Xu, Xibo Fu

**Affiliations:** ^1^ General Surgery, Dangdong First Hospital, Dandong, China; ^2^ Key Laboratory of Hepatosplenic Surgery, Department of Hepatic Surgery, The First Affiliated Hospital of Harbin Medical University, Harbin, China; ^3^ The Medical Department, Jiangsu Simcere Diagnostics Co., Ltd, Nanjing, China; ^4^ Nanjing Simcere Medical Laboratory Science Co., Ltd, Nanjing, China; ^5^ The State Key Lab of Translational Medicine and Innovative Drug Development, Jiangsu Simcere Diagnostics Co., Ltd, Nanjing, China; ^6^ Department of Gastrointestinal/Hernia and Abdominal Wall Surgery, The First Hospital of China Medical University, Shenyang, China; ^7^ Department of Hepatopancreatobiliary Surgery, Liaoning Cancer Hospital and Institute, Cancer Hospital of China Medical University, Shenyang, China

**Keywords:** hepatocellular carcinoma (HCC), immune checkpoint blockers (ICBs), combination immunotherapy, transcatheter arterial chemoembolization (TACE), traditional Chinese medicines (TCM)

## Abstract

More than half of new cases of hepatocellular carcinoma (HCC) and associated deaths occurring annually worldwide are recorded in China. Chinese patients with HCC exhibit special characteristics in terms of etiology, leading to differences in prognosis versus Western patients. In recent years, several angiogenesis inhibitors were approved, and immune checkpoint blockers (ICBs) were recommended as second-line therapy for advanced HCC. In addition, the recent success of a combination of atezolizumab with bevacizumab signals resulted in an essential change in the first-line treatment of HCC. We investigated the characteristics of patients with HCC in China and summarized the rapidly emerging relevant clinical data, which relate to the prospects and challenges associated with the use of ICBs in this setting. We further evaluated the efficacy of ICBs in Chinese patients with HCC based on data obtained from global trials, and discussed possible factors influencing the effectiveness of ICBs in patients with HCC in China. Immunotherapy offers new options for the treatment of advanced HCC, though responses varied between patients. Currently, there is a need to discover specific biomarkers for the accurate identification of patients who would more likely benefit from immunotherapy. Furthermore, investigation of patient characteristics in different countries is necessary to provide a clinical practice basis and reference value for the diagnosis and treatment of HCC.

## Introduction

According to 2018 Global Cancer Statistics, liver cancer is the sixth most common and fourth most deadly type of cancer worldwide, and hepatocellular carcinoma (HCC) accounts for 75%–85% of cases (1). HCC is very common in China, accounting for 55.41% and 53.96% of the total number of new liver cancer cases and related deaths, respectively, reported worldwide in 2018 (2). Despite advances in medicine, the 5-year survival rate of patients with HCC in China remains very low (i.e., < 13%) (3). The reasons for this poor survival rate are as follows. Firstly, most patients have both HCC and fundamental liver disease, such as viral hepatitis and cirrhosis, which complicates disease management. Secondly, HCC is usually diagnosed at advanced stages, limiting therapeutic options. Finally, there are few drugs available for the treatment of patients with advanced HCC, namely sorafenib (4, 5), lenvatinib (6), and systemic chemotherapy containing oxaliplatin (7). However, differences in background characteristics and etiology between Chinese and Western patients (8) result in a low objective response rate to treatment and limited survival benefits (9, 10). Following the failure of first-line treatment, the efficacy of second-line treatment is insufficient, and the drug accessibility is poor; hence, there is an urgent clinical need for the development of new drugs.

Immunotherapy is effective and safe for the treatment of solid tumors, such as lung cancer and melanoma. Based on the marked improvement in progression-free survival and overall survival (OS) observed in the IMbrave 150 trial (11), atezolizumab + bevacizumab (T + A) was approved by the US Food and Drug Administration as first-line treatment for advanced HCC, initiating a new era of HCC immunotherapy. Currently, clinical studies on immunotherapy for patients with HCC in China are ongoing, showing significant benefits.

In this review, we summarized recent clinical data of studies on immune checkpoint blockers (ICBs) in Chinese patients with HCC. Moreover, we analyzed differences in the efficacy of immunotherapy between Chinese and Western patients according to global results. We also discussed possible factors influencing the efficacy of immunotherapy uniquely in Chinese patients with HCC. More clinical trials tailored to national and ethnic characteristics are warranted to investigate differences in clinical results due to particular patient characteristics in different geographical regions.

## ICBs in Chinese Patients With Advanced HCC

The liver is considered an immune-tolerant organ, and HCC is a typical inflammatory-associated malignancy with a complex immune microenvironment. ICBs can stimulate immune cells to recognize and kill tumor cells by enhancing the immune response, inducing tumor-specific immunity, overcoming immune tolerance, and reactivating immune cells (12–14). These effects propose a new treatment strategy for patients with HCC, involving the use of ICBs alone or in combination with targeted therapies, or other therapies.

### ICB Monotherapy

A phase I clinical trial (NCT02742935) was initiated to evaluate the safety, pharmacokinetics, pharmacodynamics, and preliminary antitumor activity of the ICB camrelizumab (15). A total of 36 patients with advanced solid tumors were enrolled, including two patients with HCC. The data showed that 25% of the patients treated with all doses of camrelizumab had a sustained objective response. Two patients with HCC received camrelizumab (400 mg) intravenously, with an overall response rate (ORR) of 50% (1/2). Although the sample size was limited, the results of this clinical trial demonstrated the favorable antitumor efficacy of camrelizumab.

Subsequently, a multicenter, open-label, parallel-grouped, randomized phase II trial (NCT02989922) was promptly initiated to evaluate the efficacy and safety of camrelizumab in the second-line treatment of advanced HCC (16). A total of 220 eligible patients were enrolled; 217 patients were treated and included in the analysis. The patients were randomly assigned (1:1) to receive monotherapy with camrelizumab at a dose of 3 mg/kg every 2 or 3 weeks. The primary endpoints were ORR (per blinded independent central review) and 6-month OS. The results showed that, in patients pretreated with at least one line of systemic therapy, camrelizumab achieved an ORR of 14.7% (95% confidence interval [CI]: 10.3–20.2) and a 6-month OS probability of 74.4% (95% CI: 68.0–79.7). Based on this finding, camrelizumab was approved as a second-line treatment for patients with advanced HCC by the National Medical Products Administration (NMPA).

Thus far, this is the largest clinical study of immunotherapy in Chinese patients with intermediate- to advanced-stage HCC. In addition, it is the largest global clinical study of a programmed cell death 1 (PD-1) inhibitor for the treatment of advanced HCC with hepatitis B virus (HBV) infection. This investigation offers more hope for the patients with advanced HCC in the condition of few treatment options.

RATIONALE 208 (17) is a global, multicenter, phase II clinical study evaluating the efficacy and safety of tislelizumab monotherapy in patients with HCC who have previously received at least one line of systemic therapy. Patients with Barcelona Clinic Liver Cancer stage B/C, Child–Pugh grade A HCC were enrolled and treated with tislelizumab (200 mg) intravenously once every 3 weeks until they developed intolerable toxicity, expressed their desire to withdraw from the study, or did not benefit from the treatment as determined by the investigator.

The primary endpoint of the study was ORR assessed by the Independent Evaluation Committee in accordance with the Response Evaluation Criteria in Solid Tumors, version 1.1 (RECIST v1.1). This study recruited 249 patients (122 in China), including >100 who had received at least two lines of treatment prior to enrolment. The efficacy data were impressive. The ORR for the total population and second-line population was 13.3% and 13.8%, respectively. The median OS was 13.2 and 13.8 months, respectively. The disease control rate (DCR) for the general population was 53.0%, and the 12-month event-free rate was 79.2%. These data showed the advantages offered by tislelizumab in the treatment of HCC. Tislelizumab is a promising option for the second-line treatment of HCC in the future, and will also benefit numerous patients with HCC. At present, the results disclosed in this study do not compare the efficacy of tislelizumab between Chinese and Western populations; hence, we look forward to more detailed results from the follow-up of this clinical study. In addition, there is an ongoing phase III clinical trial (RATIONALE 301 study) involving a head-to-head comparison of tislelizumab with sorafenib for the first-line treatment of advanced HCC; results, from the follow-up of this study are currently awaited.

CheckMate 459 is a randomized, multicenter, phase III study of nivolumab versus sorafenib as first-line treatment in patients with advanced HCC. A total of 743 patients with advanced HCC were enrolled, and the primary endpoint was OS. Although the results did not reach the predefined threshold for statistical significance, this trial yielded some interesting and important findings. Clinical benefit was observed across predefined subgroups, including hepatitis infection status and region (Asia vs. non-Asia) (18). A similar observation was recorded in KEYNOTE-240, which involved 408 patients with advanced HCC; the coprimary endpoints in this study were OS and progression-free survival (PFS). It has been reported that benefits in OS and PFS were associated with viral status and geographical region. Particularly, OS was positively correlated with HBV infection (hazard ratio [HR] = 0.57; 95% CI: 0.35–0.94) and Asia, except for Japan (HR = 0.59; 95% CI: 0.37–0.95) (19). Notably, pembrolizumab reduced the risk of death by 45% in the Asian subgroup compared with 22% in the overall cohort. In the Asian subpopulation, the median PFS was 2.8 months versus 1.4 months in the pembrolizumab group versus the control group, respectively (HR = 0.48; 95% CI: 0.32–0.70; P < 0.0001). The median OS was 13.8 months versus 8.3 months, respectively (HR = 0.55; 95% CI: 0.37–0.80; P = 0.0009). The ORR was 20.6% versus 2.0%, respectively (treatment difference: 18.5%; 95% CI: 8.3–27.6; P = 0.0014). The median DCR was 8.6 months versus 2.8 months, respectively, while the DCR was 59.8% versus 40.0%, respectively (treatment difference: 20.1%; 95% CI: 3.1–35.7; P = 0.0101). The rate of treatment-related adverse events was 58.9% versus 48.0%, respectively. The rate of grades 3–5 treatment-related adverse events was 13.1% versus 4.0%, respectively. Notably, there was no occurrence of treatment-related deaths. These results suggested that pembrolizumab is effective and well tolerated in the second-line treatment of advanced HCC in Asian patients (20) (**Table 1**).

**Table 1:** Trials of Immune Checkpoint Blockers in Chinese Advanced Hepatocellular Carcinoma.

Source	Phase	Treatment (No.of patients)	ORR (%)	OS at 6 mo %	OS at 12 mo %	mPFS, mo	DCR (%)	mOS, mo
NCT02989922 (Second-line) Qin et al. (16)	Phase II	Camrelizumab (217)	14.7	74.4	NR	2.1	44.7	13.8
RATIONALE 208 (Second-line) M. Ducreuc et al. (17),	Phase II	Tislelizumab (249)	13.3 (RECIST v1.1)	76.4	52.6	2.7	53.0%	13.2
RESCUE (First line/second-line) Xu et al. (21)	Phase II	Camrelizumab + apatinib (190, 70 patients in the first-line setting)	34.3 (RECIST v1.1) 45.7 (mRECIST)	NR	74.7	5.7	84.62%	20.3 NE
NCT04052152 (First-line) Chen et al. (22)	Phase II	Sintilimab + Anlotinib (20)	40% (RECIST 1.1)	66.4%	NR	14.56	95%	NR
NCT04072679 Zhang et al. (23)	Phase Ib	Sintilimab + IBI305 (29 in low-dose group, 21 in high-dose group)	24.1 (low-dose) 33.3 (high-dose) (RECIST v1.1)	60.5 (low-dose) 75.8 (high-dose)	NR	NR	75.9 (low-dose) 83.3 (high-dose)	NR
ORIENT-32 (First-line) Ren et al. (24)	Phase II-III	Sintilimab + IB305 vs. Sorafenib (571)	20.5% vs 4.1% (RECIST v1.1)	NR	62.4	4.5 vs. 2.8	NR	NE vs. 10.4

NR, not reported; NE could not be evaluated. ORR, objective response rate; DCR, disease control rate. mOS, median overall survival; PFS, median progression-free survival.

### ICBs + Antiangiogenic Agents

Previous studies have indicated that antiangiogenic agents can enhance the efficacy of immunotherapy against cancer (25, 26). Thus, the combination of antiangiogenic therapy with immunotherapy can synergistically benefit patients with HCC.

A phase I trial of camrelizumab in combination with apatinib (vascular endothelial growth factor receptor 2 [VEGFR2] inhibitor) in the treatment of HCC, gastric cancer, and gastroesophageal junction cancer (NCT02942329) (27) was performed. The study included 43 patients (15 and 28 patients in phases Ia and Ib, respectively). The primary endpoints were safety and tolerability and RP2D (phase II dose) determination of apatinib in combination with camrelizumab. The efficacy endpoints included investigator-assessed ORR, DCR, PFS, and OS. Of the 16 patients with HCC, eight achieved a partial response: the ORR and DCR were 50% and 93.8%, respectively; the median OS was not reached. These results were impressive since the patients with HCC enrolled in this study were refractory patients who had previously received first-line treatment with sorafenib, reflecting the effectiveness of the combination of camrelizumab and apatinib.

Based on the results of this phase I trial, a non-randomized, open, multicenter, phase II clinical study was conducted in 25 centers in China (RESCUE) (21), involving patients with HCC who were refractory to initial treatment or first-line targeted therapy. The study included 190 Chinese patients, who received camrelizumab intravenously (200 mg [for bodyweight ≥ 50 kg] or 3 mg/kg [for bodyweight < 50 kg] every 2 weeks) + apatinib (250 mg/day, 4-week cycles). The primary endpoint was ORR assessed by the Independent Review Board according to the RECIST v1.1. At the data cutoff in June 2021 (28), in the first- and second-line treatment cohorts, the median follow-up time was 19.8 and 21.7 months; the median OS was 20.1 and 21.8 months; and the 24-month OS was 43.3% and 44.6%, respectively.

According to the baseline characteristics of the enrolled patients, >80% had a history of HBV infection, and most had advanced HCC with portal vein tumor thrombus. These findings are in line with the current situation in China, providing strong evidence for the treatment of HCC. In terms of therapeutic effect, the ORR and OS observed in the first- and second-line treatment groups undoubtedly reflect the good application prospects of the combination of camrelizumab with apatinib in the first- and second-line treatment of liver cancer. It is preferable to become a new first-line therapeutic option for HCC. The safety profile of the combination therapy was similar to that noted with camrelizumab monotherapy, and the overall toxicity was controllable and tolerated. In addition, the combination regimen can significantly reduce the incidence of reactive skin capillary hyperplasia.

Based on the above data, the combination of camrelizumab and apatinib may change the paradigm for the treatment of HCC in the future. Of note, a phase III clinical trial (NCT03764293) of camrelizumab combined with apatinib versus sorafenib in the first-line treatment of advanced HCC is currently underway.

Furthermore, another phase Ib trial of Chinese PD-1 inhibitor, sintilimab, in combination with a bevacizumab biosimilar (IBI305) for the treatment of advanced HCC was launched (NCT04072679) (23). A total of 50 patients were enrolled, and the ORR reached 33.3%. The results confirmed both the safety and efficacy of this combination regimen, paving the way for subsequent phase II/III trials.

Sintilimab + IBI305 versus sorafenib for the first-line treatment of advanced HCC (ORIENT-32 trial) was a phase III, randomized, open-label, international multicenter study (24). A total of 571 patients with unresectable or metastatic HCC who had not received systematic treatment were enrolled. The coprimary endpoints were OS and PFS, determined by the Independent Radiologic Review Committee according to the RECIST v1.1. The majority of patients (94.2%) had HBV, and 4.2% had Child–Pugh-B status chronic liver disease. The median PFS (4.5 vs. 2.8 months) and OS (not evaluable vs.10.4 months; HR = 0.57) were significantly prolonged in the sintilimab + IBI305 group versus the sorafenib group; the 1-year OS rate was 62.4% versus 48.5%, respectively. In all relevant subgroups, the OS and PFS of sintilimab + IBI305 were superior to sorafenib. The ORR, assessed by the Independent Radiologic Review Committee according to the RECIST v1.1, was 20.5% in the sintilimab + IBI305 group and 4.1% in the sorafenib group; the ORR evaluated by the RECIST v1.1 was 19.6% versus 2.9%, respectively. Among patients receiving at least one dose of the drug, the incidence of treatment-related adverse events was 88.7% (n = 380) in the sintilimab + IBI305 group and 93.5% (n = 185) in the sorafenib group. The incidence of grades 3–4 treatment-related adverse events was 33.7% and 35.7%, respectively. The combination of sintilimab with IBI305 in the first-line treatment of advanced HCC demonstrated significantly greater clinical efficacy compared with sorafenib monotherapy. Moreover, the combination immunotherapy did not increase the risk of adverse effects. Based on these impressive clinical data, on June 25, 2021, the NMPA approved the combination of sintilimab with IBI305 for the first-line systemic treatment of HCC (**Table 1**).

IMbrave 150 was an international, multicenter, clinical trial involving 501 patients with unresectable HCC who had not previously received systemic treatment; the study population included 194 patients from China (137 from the IMbrave150 Global Study and 57 from the China extension enrolment phase). Patients were randomly assigned to the T + A (Atezolizumab plus Bevacizumab) or sorafenib group in a 2:1 ratio, and the coprimary endpoints were OS and PFS assessed at an independent review facility according to the RECIST v1.1. In terms of effectiveness, the latest data showed that the global median OS in the T + A group was 19.2 months, reducing the risk of death by 34% (HR: 0.66, 95% CI: 0.52–0.85). Interestingly, the patients with HBV had longer PFS (HR = 0.47; 95% CI: 0.33–0.67) and OS (HR = 0.51; 95% CI: 0.32–0.81) (29). In the subgroup of 133 Chinese patients treated with T + A, the median OS reached up to 24 months, reducing the risk of death by 47% (HR: 0.53, 95% CI: 0.35–0.80) (30). Data from the Chinese subgroup, presented at the Liver Cancer Summit of the European Association for the Study of the Liver earlier this year, also suggested that T + A may be a more suitable option for Chinese patients (31). Specifically, the data showed that the HR value of OS in the Chinese subgroup was 0.53, and the 6-month survival rate of patients was 86.6%. These findings indicated that the efficacy was better in Chinese patients than other patients (**Table 2**), though these patients had a higher overall rate of HBV infection, extensive vessel invasion/extrahepatic metastasis, alpha-fetoprotein levels ≥ 400 ng/ml, and other adverse prognostic factors (**Table 3**). Additionally, the safety data showed that the toxicity of this regimen in the Chinese population was similar to the global population, and could be managed (**Table 2**).

**Table 2:** Comparison of Chinese population and global population data in IMbrave 150.

	IMbrave 150			
	Chinese patients		Global patients	
Treatments	Atezolizumab +bevacizumab	sorafenib	Atezolizumab + bevacizumab	sorafenib
Sample size	133	61	336	165
Median follow-up time	6.8 mo		15.6 mo*	
ORR (mRECIST)	30% ORR, 8% CR	8%ORR, 0CR	35%ORR, 12%CR*	14%ORR, 2.5%CR*
mPFS	5.7 mo	3.2 mo	6.9 mo*	4.3 mo*
	HR = 0.6*		HR = 0.65	
mOS	24 mo*	11.4 mo*	19.2 mo*	13.4 mo*
	HR = 0.53*		HR = 0.66*	
Safety	Grade 5 event, 2%; adverse event leading to withdrawal from any trial drug, 8%	Grade 3 or 4 event, 38%; Grade 5 event, 2%; adverse event leading to withdrawal from any trial drug, 2%	Grade 3 or 4 event, 62.9%; Grade 5 event, 7%; adverse event leading to withdrawal from any trial drug, 21.9%	Grade 3 or 4 event, 57.1%; Grade 5 event, 5.8%; adverse event leading to withdrawal from any trial drug, 11.5%

ORR, objective response rate; CR, complete response; OS, overall survival; PFS, progression-free survival. NE could not be evaluated. Mo, month. mPFS, median progression-free survival; mOS, median overall survival.

*we update all of the results of global data in IMbrave 150 from ASCO-GI 2021, the data in the Chinese subgroup update the mOS and OS at 12 mo; the other results were all from ESMO-ASIA 2019.

**Table 3:** Basline characteristics of Chinese population and global population in IMbrave 150.

Characteristic	Chinese		Global	
	Atezolizumab + Bevacizumab (n=133)	Sorafenib (n=61)	Atezolizumab + Bevacizumab (n=336)	Sorafenib (n=165)
Median age (range), years	57 (29-82)	60 (31-82)	64 (26-88)	66 (33-87)
Sex, male, n (%)	116 (87)	49 (80)	277 (82)	137 (83)
ECOG PS1, n (%)	55 (41)	30 (49)	127 (38)	62 (38)
Chind-Pugh class, n (%)				
A/B	132 (99)/1 (1)	61 (100)/0	333 (99)/1 (<1)	165 (100)/0
Aetiology of HCC, n (%)				
HBV/HCV/Non-viral	177 (88)/10 (8)/6 (5)	47 (77)/7 (11)/7 (11)	164 (49)/72 (21)/100 (30)	76 (46)/36 (22)/53 (32)
BCLC staging at study entry, n (%)				
A/B/C	3 (2)/15 (11)/115 (86)	1 (2)/3 (5)/57 (93)	8 (2)/52 (15)/276 (82)	6 (4)/26 (16)/133 (81)
AFP≥400ng/mL, n (%)	59 (44)	30 (49)	126 (38)	61 (37)
EHS, n (%)	91 (66)	40 (66)	212 (63)	93 (56)
MVI, n (%)	49 (37)	28 (46)	129 (38)	71 (43)
EHS and/or MVI, n (%)	111 (83)	53 (87)	258 (77)	120 (73)
Prior TACE, n (%)	71 (53)	27 (44)	130 (39)	70 (42)

AFP, α-fetoprotein; MVI, macrovascular invasion; EHS, extrahepatic spread; ECOG, Eastern Cooperative Oncology Group; HCC, hepatocellular carcinoma; TACE, Transarterial chemoembolization.

Both ORIENT32 and IMbrave150 were approved in China, so which one is the better choice for the Chinese HCC patients? In ORIENT-32, sintilimab was PD-1 antibody, while atezolizumab was PD-L1 in IMbrave150. Moreover, the characteristics of patients included in these two studies differed. Patients who enrolled in ORIENT-32 had poor performance status scores; a high proportion of these patients also had adverse prognostic factors, such as HBV infection, extrahepatic metastasis, and alpha-fetoprotein levels ≥ 400 ng/ml (24). Therefore, the results of ORIENT-32 are more meaningful for the treatment of HCC in China, as it reflected the characteristics of Chinese patients with HBV-related HCC. Particularly, the impaired liver function due to cirrhosis impairs the efficacy of sorafenib-targeted therapy in Asian patients with HCC, and at present there is an unmet need for the treatment of such patients. In addition, the two treatment regimens led to similar benefits in OS and PFS; however, the ORR of sintilimab + IBI305 was slightly lower than T + A combination, which may be caused by the coronavirus disease in 2019. Besides, the T + A combination is more expensive, whereas sintilimab + IBI305 is cost effective, highly accessible, and covered by domestic medical insurance schemes in China. In summary, sintilimab + IBI305 may be a more suitable option for first-line treatment in Chinese and other Asian patients.

In addition, the use of sintilimab + anlotinib as first-line therapy in patients with advanced HCC was investigated in a single-arm phase II study(NCT04052152). Until January 15, 2021, 20 patients (all with HBV infection, 18 men and two women) had enrolled in the clinical trial. The primary endpoints (i.e., safety and ORR) were evaluated after first-line treatment with sintilimab (200 mg, intravenously, D1) + anlotinib (12 mg, orally, once daily, D1–14) every 3 weeks. The phase results of this clinical trial were reported at the 2021 American Society of Clinical Oncology Annual Meeting (22): the ORR was 40.0% (8/20); one, seven, and 11 patients had complete response, partial response, and stable disease, respectively. The most common treatment-related adverse events were grades 1–2. According to these preliminary results, the combination of sintilimab and anlotinib showed promising clinical efficacy with manageable toxicity for the first-line treatment of advanced HCC. This treatment regimen further enriches the available options for the first-line treatment of patients with advanced HCC. We look forward to the complete results of this study.

Numerous multicenter, phase III, clinical trials are currently ongoing (**Table 4**); we hope that these investigations will provide more clinical data to support the application of ICBs.

**Table 4:** Ongoing Randomized Phase III Trials of Immune Checkpoint Blockers in Chinese HCC.

Clinical Trials.gov identifier	Study title	Phase, setting	Treatment (No. of patients)	Primary endpoint
NCT03412773 (First-line)	Phase 3 Study of Tislelizumab Versus Sorafenib in Participants With Unresectable HCC	Phase III, unresectable HCC	Tislelizumab (PD-1 mAb) vs Sorafenib (TKI) (674)	OS
NCT04720716 (First-line)	A Study to Compare the Effectiveness and Safety of IBI310 Combined With Sintilimab Versus Sorafenib in the First-line Treatment of Advanced HCC	Phase III, advanced	Sintilimab (PD-1 mAb) + IBI310 (CTLA-4 mAb) vs Sorafenib (TKI) (490)	OS, ORR
NCT04723004 (First-line)	Evaluate the Safety and Efficacy of Toripalimab Combined With Bevacizumab Versus Sorafenib Therapy for HCC	Phase III, advanced	Toripalimab (PD-1 mAb) + bevacizumab (TKI) vs Sorafenib (280)	PFS, OS
NCT04523493 (First-line)	Phase III Study of Toripalimab (JS001) Combined With Lenvatinib for Advanced HCC	Phase III, advanced	Toripalimab (PD-1 mAb) + lenvatinib (TKI) vs Placebo + lenvatinib (486)	PFS, OS
NCT04344158 (First-line)	A Phase III Clinical Trial of AK105 Injection Combined With Anlotinib Hydrochloride Capsules Versus Sorafenib in Subjects With Advanced Hepatocellular Carcinoma (HCC)	Phase III, advanced	AK105 (PD-1 mAb) + anlotinib (TKI) vs Sorafenib Tosylate Tablets (648)	OS
NCT04560894 (First-line)	SCT-I10A Plus SCT510 Versus Sorafenib as First-Line Therapy for Advanced Hepatocellular Carcinoma (HCC)	Phase II/III, advanced	SCT-I10A (PD-1 mAb) + SCT510 (TKI) vs Sorafenib (621)	PFS, OS
NCT04194775 (First-line)	A Study of CS1003 in Subjects With Advanced Hepatocellular Carcinoma	Phase III, advanced	CS1003 (PD-1 mAb) + lenvatinib (TKI) vs CS1003 Placebo + lenvatinib (525)	PFS, OS
NCT04465734 (First-line)	A Clinical Study to Compare the Efficacy and Safety of HLX10 in Combination With HLX04 Versus Sorafenib as the First-line Treatment in Patients With Locally Advanced or Metastatic HCC	Phase III, advanced	HLX10 (PD-1 mAb) + HLX04 (TKI) vs Sorafenib (TKI) (477)	PFS, OS
NCT03755791 (First-line)	Study of Cabozantinib in Combination With Atezolizumab Versus Sorafenib in Subjects With Advanced HCC Who Have Not Received Previous Systemic Anticancer Therapy	Phase III, advanced	Atezolizumab (PD-L1 mAb) + cabozantinib (TKI) vs Cabozantinib vs Sorafenib (740)	PFS, OS
NCT04039607 (First-line)	A Study of Nivolumab in Combination With Ipilimumab in Participants With Advanced Hepatocellular Carcinoma	Phase III, advanced	Nivolumab (PD-1 mAb)+ Ipilimumab (CTLA-4 mAb) vs Sorafenib/lenvatinib (650)	OS
NCT03298451 (First-line)	Study of Durvalumab and Tremelimumab as First-line Treatment in Patients With Advanced Hepatocellular Carcinoma	Phase III, advanced	Durvalumab (PD-L1 mAb) vs Durvalumab + tremelimumab (CTLA-4 mAb) vs Sorafenib (1504)	OS
NCT04665609	Thermal Ablation Combined With Anlotinib and TQB2450 Solution for HCC	Phase III, advanced	TQB2450 solution (PD-L1 mAb) + Anlotinib (TKI) vs TQB2450 Solution (80)	ORR
NCT04229355	DEB-TACE Plus Lenvatinib or Sorafenib or PD-1 Inhibitor for Unresectable Hepatocellular Carcinoma	Phase III, unresectable HCC	DEB-TACE + Sorafenib (TKI) vs DEB-TACE + lenvatinib (TKI) vs DEB-TACE + PD-1 inhibitor (90)	PFS
NCT04712643	A Study of TACE Combined With Atezolizumab Plus Bevacizumab or TACE Alone in Patients With Untreated Heaptocellular Carcionma	Phase III, untreated HCC	Atezolizumab (PD-L1 mAb) + bevacizumab (TKI) + TACE vs TACE (342)	PFS, OS
NCT04246177	Safety and Efficacy of Lenvatinib (E7080/MK-7902) With Pembrolizumab (MK-3475) in Combination With Transarterial Chemoembolization (TACE) in Participants With Incurable/Non-metastatic Hepatocellular Carcinoma (MK-7902-012/E7080-G000-318/LEAP-012)	Phase III, incurable/non-metastatic	Pembrolizumab (PD-1 mAb) + Lenvatinib (TKI) + TACE vs Oral Placebo + IV Placebo + TACE (950)	PFS, OS
NCT03778957	A Global Study to Evaluate Transarterial Chemoembolization (TACE) in Combination With Durvalumab and Bevacizumab Therapy in Patients With Locoregional Hepatocellular Carcinoma	Phase III, locoregional HCC	TACE + Durvalumab (PD-L1 mAb) vs TACE + Durvalumab + Bevacizumab (TKI) vs TACE + Placebos (710)	PFS

ORR, objective response rate; OS, overall survival; PD-1, programmed cell death protein 1; PD-L1, programmed death 1 ligand 1; mAb, monoclonal antibody; PFS, progression-free survival; TACE, transarterial chemoembolization; TKI, tyrosine kinase inhibitor.

### ICBs Combined With Interventional Therapy

Apart from the aforementioned immunotherapy methods, the combination of ICBs with interventional therapy is another treatment strategy. The most commonly used local treatments or interventional therapies include transcatheter arterial chemoembolization (TACE), transhepatic artery radioembolization (TARE), and hepatic artery infusion chemotherapy (HAIC).TACE can cause tumor necrosis, which promotes the release of tumor-associated antigens and neoantigens. Thus, theoretically, the combination of TACE with ICBs may exhibit synergy, thereby enhancing the antitumor immune effect (32–35). Preliminary study results of nivolumab combined with drug-eluting bead-TACE, reported in the 2020 Gastrointestinal Cancers Symposium of the American Society of Clinical Oncology (36), have confirmed the safety and efficacy of this regimen. A preliminary retrospective case series demonstrated that TARE combined with immunotherapy was safe and effective, and the combination therapy may improve PFS and OS in patients with liver metastases from uveal melanoma (37). A case report showed a significant response following consecutive treatment with TARE, sorafenib, and nivolumab in a patient with metastatic HCC (38). This finding suggested that TARE, sorafenib, and nivolumab may exert a synergistic effect on the immune response to HCC. However, large-scale, prospective clinical trials are warranted to validate these results. A retrospective study evaluated the efficacy of HAIC or HAIC combined with anti-PD-1 immunotherapy (HAICAP) in patients with advanced HCC. The investigators found that patients treated with HAICAP had significantly better treatment response and survival benefits than those treated with HAIC (39).

Another retrospective study (40) compared the efficacy of lenvatinib + toripalimab (PD-1 monoclonal antibody) + HAIC (LeToHAIC, n = 71) with that of lenvatinib (n = 86) in patients with advanced HCC. The results revealed that LeTOHAIC was associated with longer PFS (11.1 vs. 5.1 months, respectively; HR = 0.48, 95% CI: 0.33–0.7, P < 0.001) and OS (< 11 months; HR = 0.4, 95% CI: 0.24–0.66, P < 0.001). The DCR (90.1% vs. 72.1%, respectively, P = 0.005) and ORR (RECIST: 59.2% vs. 9.3%, respectively, P < 0.001; modified RECIST: 67.6% vs. 16.3%, respectively, P < 0.001) in the LeTOHAIC group were superior to those recorded in the lenvatinib monotherapy group. Immuno-targeted therapy could eradicate residual tumor lesions after HAIC, and patients achieved longer survival. A real-world study of HAIC combined with anti-PD-1 immunotherapy and tyrosine kinase inhibitors (TKIs) for advanced HCC showed that HAIC + anti-PD-1 antibody + oral TKIs were effective and safe. In this study, ORR (63% vs. 36.7%–46%, respectively) and median PFS (10.6 vs. 5.5–9.3 months, respectively) were higher than those reported in a previous study of anti-PD-1 antibody combined with TKI for unresectable HCC (41).

Although combined immunotherapy has entered an era of blooming flowers, and the efficacy of advanced HCC patients has been greatly improved, there are still many issues worth considering and exploring. Firstly, targeted therapy combined immunotherapy has brought gratifying results, suggesting that we have various systemic therapies deserve further investigation, including but not limited to immune checkpoint inhibitors + antiangiogenic agents or tyrosinase inhibitors (TKIs). New targets, new drugs with different mechanisms, and new combination regimens are all worth trying, for example, a stronger combination: PD-1 inhibitor + targeted therapy + TACE, etc. Secondly, we have revealed good efficacy of combined immunotherapy in advanced patients, and the application of immunotherapy combination models in early and mid-stage HCC needs to be further explored, such as immunotherapy combined targeted therapy for the perioperative treatment of HCC. Finally, we need to explore the dominant populations that benefit from immunotherapy and look for biomarkers that can effectively predict the efficacy. Numerous larger prospective studies are currently underway in China, investigating the optimal combination therapy; the results of these studies are anticipated (**Table 4**).

## Chinese Patients With HCC: Characteristics and Their Correlation With Immunotherapy

HCC in China is characterized by distinct etiology, biological events at the molecular level, and prognosis, thus requiring a specific treatment strategy. Firstly, >70% of patients with HCC in China have HBV infection (8); it is thought that these patients who are more prone to develop progressive disease and have poorer prognoses than those with hepatitis B virus (HCV) infection (9, 10). Secondly, there are significant differences in the mutation landscape between Chinese and Western patients. Jia Fan et al. reported that the mutation frequencies in axin 1 (AXIN1), tuberous sclerosis complex subunit 2 (TSC2), SWI/SNF related, matrix associated, actin dependent regulator of chromatin, subfamily a, member 2 (SMARCA2), ATRX chromatin remodeler (ATRX), and lysine methyltransferase 2C (KMT2C) were relatively higher, whereas that of catenin beta 1 (CTNNB1) was significantly lower in the Chinese HBV-positive HCC cohort versus the HBV-positive HCC subgroup from The Cancer Genome Atlas (42). Thirdly, approximately 60%–70% of patients with HCC were diagnosed at intermediate-to-advanced stages of the disease, and most had undergone TACE as first-line palliative treatment instead of surgical excision (43). Finally, traditional Chinese medicine (TCM) is widely used in China, and is effective in improving the quality of life, modulating immune functions, preventing recurrence and metastasis, delaying tumor progression, and prolonging survival in patients with cancer (44–49). Almost 80% of patients with HBV in China are treated with TCM (50). Moreover, aristolochic acid (AA), a compound used in TCM, can cause genomic damage and particular gene mutations in liver cells (51–54). These effects occurred in 78% and 47% of Taiwanese and Chinese patients, respectively; these proportions are markedly higher than those reported in Western countries (55).

### HBV Infection

HBV infection could lead to changes in the liver immune microenvironment. It causes immunosuppression, and leads to peripheral immune tolerance with the development of chronic infection. Finally, it mediates tumorigenesis due to impaired immune surveillance (56). In chronic viral hepatitis, immunosuppressive checkpoints, including PD-1/PD-L1, cytotoxic T-lymphocyte associated protein 4 (CTLA4), and T cell immunoglobulin and mucin domain 3 (TIM3), play an important role in immunosuppression by downregulating the T cell response (57).

Lim et al. (58) reported that regulatory T cells and CD8+ resident memory T cells were enriched in HBV-related HCC. Regulatory T cells and memory T cells obtained from patients with HBV-related HCC expressed more PD-1 and were functionally more immunosuppressive and exhausted than those of nonvirus-related HCC. This immunosuppression by PD-1 + regulatory T cells could be reversed by anti-PD-1 blockade. The microenvironment of HBV-related HCC is more immunosuppressive and exhausted than that of nonviral-related HCC. Despite the lack of comparison with HCV-HCC, these results had important implications in the management of HBV-HCC.

The results of two studies with small sample sizes, namely CheckMate 040 (59) and KEYNOTE-224 (60), showed that the efficacy of ICBs was not affected by HBV or HCV infection. Nevertheless, further data from the CheckMate 459 and KEYNOTE-240 studies revealed that clinical benefit was associated with the infection status. In KEYNOTE-240, the ORR associated with pembrolizumab or placebo at the time of the final analysis was 18% and 4%, respectively (one-sided P < 0.001). In the Asian subgroup, the ORR was 21% and 2%, respectively (P < 0.001). Notably, the proportion of patients with HBV infection status was markedly higher in the Asian subgroup versus the overall group (51% vs. 25%, respectively). In the corresponding subgroup analysis of data from the IMbrave 150 study, patients with HBV-HCC may benefit more than those with nonviral HCC. Notably, T + A significantly extended the median PFS of patients with HBV-positive HCC compared with sorafenib; however, this effect was not observed in patients with nonviral HCC (median PFS, HBV-positive HCC: 6.7 vs. 2.8 months, respectively; nonviral HCC: 7.1 vs. 5.6 months, respectively) (61). Additional prospective clinical trials are warranted in the future to confirm the relationship between the infection status and efficacy of ICBs.

### Gene Mutation Landscapes

Fan et al. (42) reported that the mutation frequencies in genes AXIN1, TSC2, SMARCA2, ATRX, and KMT2C were relatively higher, whereas that of CTNNB1 was significantly lower, in Chinese HCC patients with HBV infection cohort than in the HBV-positive HCC subgroup from The Cancer Genome Atlas. AXIN1, TSC2, ATRX, and KMT2C genes were positively correlated with immunotherapy. TSC2 and mechanistic target of rapamycin complex 1 (mTORC1) played specific roles in the induction of antitumor immunity. TSC2-deficient tumors behaviors T cell exhaustion and response to anti-PD-1/anti-CTLA4 immunotherapy (62). ATRX mutation may be a predictive biomarker for the efficacy of ICBs in female patients with gastric cancer (63). Members of the KMT2 family (e.g., KMT2A, KMT2C, and KMT2D) exhibit a high mutation rate in nonsmall cell lung cancer tumors (64, 65), and these mutations were associated with higher tumor mutational burden (TMB) and PD-L1 expression, as well as higher PD-L1+/TMB-high proportions. Importantly, nonsmall cell lung cancer patients with tumor protein p53 (TP53)/KMT2C comutations who underwent treatment with ICBs had significantly longer PFS and greater durable clinical benefit (66). CTNNB1 is one of the most common mutant genes in HCC, which encodes a β-catenin protein and is involved in the regulation of the Wnt signaling pathway. Recent studies have shown that the activation of Wnt/CTNNB1 signaling can promote the immune escape of liver tumors, leading to the development of resistance to PD-1 immunotherapy (67).

### TACE

TACE is closely related to immunotherapy; for example, TACE can rapidly “starve” the tumor by blocking the blood supply, while the necrotic tumor tissue will further stimulate the body’s high T cell response, revealing a synergistic mechanism (35, 68, 69). In other words, TACE kills HCC cells and induces the release of tumor-associated antigens, thereby enhancing the tumor-specific CD8+ T cell response. TACE can significantly increase the CD4/CD8 ratio (70) and number of T helper 17 cells (71), as well as improve the immune microenvironment of the liver to a certain extent (72) which subsequently transforms cold tumors into hot tumors. Nevertheless, TACE increased the expression of PD-1 and PD-L1 in HCC (73, 74), potentially inhibiting antitumor immunity. Therefore, the combination of TACE and ICBs may be a promising therapeutic option for optimizing tumor response and downtime therapy. However, in all clinical trials of immune monotherapy conducted thus far (i.e., KEYNOTE-224, KEYNOTE-240, CheckMate 040, CheckMate 459), TACE was not included in the subgroup analysis. Therefore, there is no evidence regarding the effect of TACE on the efficacy of immune monotherapy. In the phase 2–3 ORIENT-32 study (24), the researchers found that patients could benefit from treatment regardless of the use of TACE: TACE (OS, HR = 0.59, 95% CI: 0.46–0.76; PFS, HR = 0.59, 95% CI: 0.41–0.84); no TACE (OS, HR = 0.48, 95% CI: 0.33–0.70; PFS, HR = 0.59, 95% CI: 0.38–0.91). Therefore, the potential contribution of TACE to immunotherapy remains to be further investigated.

### TCM and AA

Long-term clinical practice has proven that treatment of HCC with TCM is effective in improving symptoms, delaying disease recurrence, reducing toxicity, increasing efficacy, and prolonging survival. At present, there is rich practical experience accumulated with regard to many Chinese patent medicines used in the treatment of primary liver cancer. For example, Huaier granules (47), Jinlong capsules (48), Chinese herbal injections (45), and elemene (44) have been included in the 2020 Chinese Society of Clinical Oncology guidelines for the diagnosis and treatment of primary liver cancer. In addition, the “Guidelines for the Diagnosis and Treatment of Chinese Medicine Tumors,” that are in line with international standards, are consistent with modern clinical practice (75). Owing to its advantages (i.e., strengthening the body, enhancing the immune system, and inducing few toxic reactions), TCM plays a complementary role in improving the symptoms, immune function, and the quality of life of patients with liver cancer. For example, Huaier granules can regulate the body’s immunity by stimulating the release of cytokines, and can also result in antitumor responses by inducing cell cycle arrest and inhibiting tumor angiogenesis. Elemene can induce apoptosis in liver cancer cells, block the cycle of liver cancer cells, and inhibit the proliferation, invasion, and metastasis of liver cancer cells. It can also inhibit angiogenesis by liver cancer cells, and regulate immune function. Jinlong capsules can reduce tumor angiogenesis, prevent recurrence and metastasis, improve immunity, and relieve pain, fatigue, and symptoms of the digestive tract. In conclusion, the regulatory effect of TCM on the human immune system cannot be ignored.

AA and its derivatives are components of numerous traditional medicines that have been used for thousands of years, particularly in Asian countries (76). Since the recognition of the nephrotoxicity and carcinogenicity of AA, the US Food and Drug Administration and the regulatory agencies of some other countries have issued warnings to prohibit the use and import of products containing *Aristolochia* (77). Nonetheless, in China and some Asian countries, herbal preparations and products containing *Aristolochia* and *Asarum* continue to be widely used. The role of AA in liver cancer remains controversial. Although many studies have shown that AA can cause liver damage and is a risk factor for HCC, there are also research results indicating that the role of AA needs to be reexamined (78). It is established that higher TMB levels and neoantigen load indicate a potential benefit from immunotherapy, such as ICBs (79). Fan et al. (42) found that the levels of TMB in AA-signature-containing HCC tumors were two-fold higher than those detected in non-AA HCC tumors. Moreover, the predicted neoantigen counts were more than two-fold higher in HCC tumors with an AA signature. They harbored significantly denser infiltrating CD8+ T cells (42) and exhibited higher expression of PD-L1, which were particularly related to tumor microenvironmental immune tolerance (80). It is suggested that HCC patients with the AA signature may benefit from immunotherapy.

Regrettably, Chinese and international clinical trials have not included the use of TCM in the subgroup analysis. Even in the ORIENT-32 clinical trial, patients with HCC who had received TCM within 2 weeks were excluded from the trial. Based on the existing research results, we cannot analyze the impact of TCM on ICBs in the real-world setting.

In addition to the factors mentioned above related to immunotherapy alone, the combination of treatments can affect immunotherapy, which also warrants further study. For example, the HBV virus can induce genetic mutations and regulate the expression of genes (81), such as telomerase reverse transcriptase (TERT) (82) and TP53 (83, 84), which may be indirectly related to immunotherapy. Some investigators found that TCM may improve immune response in patients with HCC undergoing TACE (85). It is suggested that the combination of interventional therapy and TCM may promote the immune response of patients with unresectable HCC. Moreover, studies have shown that the hepatic viral infection status, principally HBV infection, is a beneficial factor in the treatment of HCC patients with Chinese medicine combined with TACE (HR = 0.67; 95% CI: 0.53–0.84) (47). However, there is a lack of relevant evidence, and additional randomized controlled clinical trials are warranted.

## Conclusion

Currently, there are several options for the first-line treatment of HCC, including targeted drugs, immunotherapy + antivascular agents. According to two phase III studies, the combination of ICBs + antivascular agents is the preferred choice. Among them, sintilimab + IBI305 may be more suitable for Chinese patients with HCC. However, Challenges of immunotherapy include the selection of suitable patients and subsequent treatment after disease progression. Therefore, further investigation is needed.

PD-1 monotherapy is also an option for patients with TKI intolerance or contraindications to bevacizumab. In addition, the combination of tislelizumab, camrelizumab, and other ICBs with targeted therapy, chemotherapy, local therapy, and other immunotherapies is also being explored. Future research results are expected to provide diversified options for the treatment of HCC. Concerning second-line treatment of HCC, the current results are based on phase II clinical studies of immunomonotherapy, and the results of phase III studies are expected in the future.

Chinese HCC patients exhibit different characteristics compared with Western patients, such as HBV infection, exposure to aflatoxin B1 (86). Additionally, a considerable number of patients received TCM. Furthermore, the genetic mutations differ between these populations. These characteristics influence the immune microenvironment of patients, thereby affecting the efficacy of immunotherapy. Therefore, it is necessary to design clinical study specific to the Chinese HCC patients. Besides, in the large, global, clinical trials, further detailed subgroup analyses should be conducted to obtain more accurate results. Such data would provide more scientific and specific reference value for clinical guidance.

## Author Contributions

YW and HL, investigation and original draft preparation. XY, TG and TS, writing–original draft, review, and editing. HX and XF, conceptualization, investigation, supervision, writing–original draft, review, and editing. All authors contributed to the article and approved the submitted version.

## Conflict of Interest

Author XY, TG and TS were employed by Jiangsu Simcere Diagnostics Co., Ltd and Nanjing Simcere Medical Laboratory Science Co., Ltd.

The remaining authors declare that the research was conducted in the absence of any commercial or financial relationships that could be construed as a potential conflict of interest.

## Publisher’s Note

All claims expressed in this article are solely those of the authors and do not necessarily represent those of their affiliated organizations, or those of the publisher, the editors and the reviewers. Any product that may be evaluated in this article, or claim that may be made by its manufacturer, is not guaranteed or endorsed by the publisher.

## Abbreviations

AA, Aristolochic acid; DCR, Disease control rate; HAIC, Hepatic artery infusion chemotherapy; HBV, Hepatitis B virus; HCC, Hepatocellular carcinoma; HR, Hazard ratio; ICB, Immune checkpoint blockers; NMPA, National Medical Products Administration; ORR, Overall response rate; OS, Overall survival; PFS, Progression-free survival; TCM, Traditional Chinese medicine; TKI, Tyrosine kinase inhibitors; TMB, Tumor mutational burden; VEGF, Vascular endothelial growth factor.
